# LUPUS ANTICOAGULANT AND LEG ULCERS IN SICKLE CELL ANEMIA

**DOI:** 10.4103/0019-5154.55635

**Published:** 2009

**Authors:** Edeghonghon E Olayemi, Godwin N Bazuaye

**Affiliations:** *From the Department of Haematology, University of Ghana Medical School, Accra, Ghana, University of Benin, Benin City, Nigeria.*

**Keywords:** *Sickle cell anemia*, *leg ulcers*, *lupus anticoagulant*, *hemolysis*

## Abstract

**Background:**

The cause of chronic leg ulcer (CLU) in patients with sickle cell anemia (SCA) is unknown; it has been attributed to hypercoagulability associated with the disease. Recently, it has been suggested that a subset of SCA patients may be prone to developing CLU and that hemolysis may be an underlying factor in the development of CLU. The lupus anticoagulant (LA) is an antiphospholipid antibody (aPLa), these antibodies have been described in patients with SCA.

**Aims:**

This study was designed to determine the frequency of LA in SCA patients with CLU compared with those without CLU.

**Materials and Methods:**

Study design is a descriptive, cross-sectional one. Thirty-three SCA patients with CLU and 33 patients without CLU were screened for the presence of LA using the Kaolin clotting time (KCT), which is an important assay, and Kaolin clotting time index was calculated. Means were compared using the students' t test, proportions were compared using the chi-square test, level of significance was set at 0.05, odds ratio was calculated.

**Results:**

About 18.18% of patients with CLU had LA, compared with 6.06% among controls (*P* < 0.05); odds ratio was 3.44 (95% CI 0.64 – 18.51).

**Conclusions:**

We conclude that SCA patients with CLU may be more likely to develop LA, and this may be related to the degree of hemolysis. Further studies are required to find out if CLU and LA are a result of hemolysis or if LA is responsible for the higher incidence of hemolysis and CLU found among these patients.

## Introduction

Sickle cell anemia (SCA) is a common genetic disease,[1] occurring with the greatest frequency in tropical Africa with a heterozygote frequency of about 20–40%.[2] The sickle cell gene is found to a lesser extent in the Middle East, Greece, and some regions of India.[3]

There is a great variation in the frequency and severity of symptoms and complications in individuals who have SCA and this is only partially explained by genetic and environmental factors.[4] The rigidity of the sickled red cells, which shortens their life span, leading to a chronic hemolytic anemia and occlusion of small blood vessels by sickled red cells that cause ischemia or infarction of distal organs[4] are believed to be responsible for most of the symptoms and signs seen in SCA.

Chronic leg ulceration (CLU) is a common complication of SCA, it occurs around the ankle[5,6] and is rare among young children.[7] Low-steady-state hemoglobin concentration is associated with a higher incidence of ulcer formation in SCA.[7] In Nigeria, a prevalence rate of 7.5% has been reported among SCA patients.[8] Recurrence rates are high and ranged from 71.4% in Lagos, Nigeria[5] to 96% in Accra, Ghana.[6] The cause of CLU in SCA patients is not clearly known, in the past the presence of leg ulcers has been attributed to the hypercoagulable state found in the disease. More recently, it has been suggested that there may be a subset of SCA patients who are prone to developing leg ulcers[9,10] and that hemolysis may be an underlying factor in the development of CLU, as they are also seen in the presence of other chronic hemolytic states such as thalassaemia and hereditary spherocytosis.[11–13]

The lupus anticoagulant (LA) is an antiphospholipid antibody (aPLa), which prolongs phospholipid – dependent coagulation tests by interfering with the coagulation reactions dependent on protein-phospholipid complexes *in vitro*.[14] Antiphospholipid antibodies have been associated with the presence of leg ulcers and these antibodies have also been described in patients with SCA. The common aPLa are the anticardiolipin antibodies (ACA) and lupus anticoagulant (LA).

Hypercoagulable states have been associated with skin ulceration.[15] The pathophysiology of venous, arterial, and arterio-venous leg ulcers remains complex, suggesting several coagulation perturbations.[16]

Hemolysis has been associated with the pathogenesis of aPLa in SCA patients. Repeated sickling has been shown to produce a disruption and rearrangement of the red cell membranes.[17] The exposure of negatively charged phospholipids may result in the formation of antibodies against these cell membrane constituents.[18]

Antiphospholipid antibodies show an affinity for negatively charged phospholipids, which are located on the inner leaflet of the bimolecular lipid membrane of platelets.[19] As the cell membranes of platelets and red blood cells have a similar phospholipid composition and distribution,[20] an immune reaction may be anticipated in diseases characterized by the perturbation and destruction of red cell membranes as in SCA.[18]

Furthermore, in patients with a high concentration of irreversibly sickled cells, such as SCA, antibodies may be formed through chronic exposure to negatively charged phospholipids.[18] Kucuk *et al*. have shown that aPLa formation can be induced in mice by phospholipid in a hexagonal II phase, but not by phospholipid in a bi-layer phase[21] Sickle red cell membranes have increased hexagonal II phase content. They also showed that up to 68% of sickle cell disease patients had aPLa, the increased aPLa in these patients is comparable with the concept that aPLa formation is associated with structural changes in the red cell membrane and that such changes occur in patients with SCA.

Leg ulcers occur in both SCA patients and in patients with antiphospholipid syndrome with the presence of LA.[22] This may be due to the predisposition to microvascular thrombosis in both disorders. This study was designed to determine if LA was more frequent in SCA patients with chronic leg ulcers (CLU) compared with those without CLU. We hypothesize that if severity of hemolysis identifies a subphenotype of SCA patients prone to developing CLU and hemolysis also plays a prominent role in development of aPLa, then patients with SCA and CLU should have a higher frequency of LA which is an aPLa, than an SCA patient without CLU.

## Materials and Methods

Thirty-three adult SCA patients with CLU seen over a period of 1 year, at the medical outpatients unit of University of Benin Teaching Hospital (UBTH), who gave informed consent, were screened for the presence of LA using the kaolin clotting time (KCT). For Subjects with a prolonged KCT, the KCT index (KCTI) was calculated, KCTI ≥ 1.2 was taken as positive for LA.

For statistical purposes, the results of these subjects were compared with 33 controls who had SCA without leg ulcers. Test subjects and controls were matched for age and sex. All 33 SCA patients with CLU in our study had leg ulcers for at least 6 months but less than a year. The study was approved by the UBTH ethics committee.

### Sample collection

Six and a half millilitres of venous blood was collected with minimum of stasis into sterile, disposable plastic syringes.

Out of this 4.5ml of blood was mixed with 0.5 ml of 0.129M trisodium citrate in a ratio of 1 part anti-coagulant to 9 parts of blood. Platelet poor plasma was prepared by centrifuging the citrated blood twice in a bench centrifuge at 2,500g for 15min at room temperature. The separated plasma samples were then preserved on ice and were analyzed within 2h. The remaining 2ml of blood was put in an EDTA bottle for platelet count, packed cell volume, and reticulocyte count. Normal pooled plasma was prepared from 5 healthy subjects who do not have coagulation defect(s). Plasma of volunteers used to prepare normal pooled platelet poor plasma were screened to ensure that they had normal prothrombin time (PT), kaolin clotting time (KCT), and platelet count.

### Procedures

Kaolin clotting time (KCT) was performed as described previously[23,24] by pre-incubating 0.2ml of citrated plasma with 0.1 ml Kaolin suspension 20g/l tris buffer at a pH 7.4 for 3min at 37° C, 0.2 ml of 0.025 M calcium chloride was then added. The time from the addition of calcium chloride to the formation of a clot was recorded; the procedure was carried out in duplicates for each sample and the average was taken as the clotting time. Plasma that had prolonged KCT were subjected to mixing studies using the KCT on prolonged plasma (PP) and normal plasma (NP) in the following proportions of NP/PP 100/0, 90/10, 80/20, 50/50, 20/80, 10/90, and 0/100 as earlier described.[23]

The KCT index, which is the ratio of KCT at 20% prolonged plasma to KCT at 100% normal plasma, of greater than or equal to 1.2, was taken to signify the presence of LA.[24]

KCT (80% normal plasma: 20% prolonged plasmaKCT 100% normal plasma=≥1.2

Platelet count, packed cell volume, and reticulocyte counts were performed as previously described.[25]

### Statistical analysis

Data collected in this study were analyzed on a computer with the statview for windows software version 5.0.1. Means were compared using the Students'test, while proportions were compared using the chi-square test; level of significance was taken as *P* < 0.05. The odds ratio was also calculated. The average values are presented as mean ± SD unless otherwise stated.

## Results

A total of 33 SCA patients with CLU and the same number of SCA patients without CLU were studied. Six (18.18%) patients with CLU had LA, compared with 2 (6.06%) among controls (*P* < 0.05); odds ratio was 3.44 (95% CI 0.64-18.51). Mean KCT was 97.58 ± 30.10 s for subjects with CLU and 80.82 ± 17.24 s for those without CLU (*P*<0.05). (Normal KCT in our laboratory is 60-110 secs[26]).

Among those with LA, mean KCT was 134.67 ± 25.98 s for those with CLU and 126.50 ± 4.95 s in those without CLU. Among the six subjects with LA, 4 (66.67%) were males and both controls with LA were also male. Mean PCV was lower in patients with CLU than those without 21.73 ± 4.52 versus 23.30 ± 4.43 though this was not statistically significant. On the other hand, patients with CLU had a higher platelet count than controls. Again this was not statistically significant. There was no significant difference in mean reticulocyte counts between test and control subjects: 12.67 ± 5.00 and 10.78 ± 3.46, respectively [Table 1]. Among the test subjects, there were 23 (69.7%) females compared with 24 (72.72%) among the controls. Mean age was 22.21 ± 4.55 years for test subjects and 22.27 ± 3.71 years for controls, there was no significant difference in the mean age.

**Table 1:** Hematological parameters in cases of SCA with leg ulcers and without leg ulcers

Parameter	SCA with leg ulcer	SCA without leg ulcer
Mean PCV (%)	21.73 ± 4.52	23.30 ± 4.43
Mean platelet count	373,757.58 ± 85,095.55	352363.64 ± 100,046.81
Mean reticulocyte count (%)	12.67 ± 5.00	10.78 ± 3.46
Mean kaolin clotting time (seconds)	97.58 ± 30.10*	80.82 ± 17.24
KCT in patients with LA (seconds)		
Lupus anticoagulant (%)	18.18*	6.06

* *P* < 0.05, SCA: Sickle cell anemia

## Discussion

LA and other aPLa have been previously described in patients with SCA, their presence has been attributed to perturbations in the red cell Membranes, which may be exacerbated by recurrent sickling and hemolysis in SCA.[18,21] The results of our studies suggest that SCA patients with leg ulcers are more likely to develop LA than those without leg ulcers. This is further reflected based on the fact that subjects with CLU had a significantly longer KCT than those without CLU. Although previous studies have suggested that a higher degree of hemolysis may define a sub-phenotype of SCA that is more likely to develop CLU,[10] our study did not confirm this observation. Despite the fact that subjects in our study with leg ulcers had a higher reticulocyte count than those without CLU, this was not statistically significant, though we did not exclude factors such as renal impairment and folate deficiency that may impair reticulocyte response to hemolysis in SCA.

Although our results suggest that SCA patients with LA and possibly other aPLa are more prone to develop leg ulcers, it is difficult to conclude that the presence of LA was the sole cause of CLU or if they arose primarily as a result of the SCA. These results are also in agreement with earlier observations that only a subset of SCA patients develop leg ulcers. It is also possible that these same subsets are more likely to have aPLa.

As there is no evidence of ulcers in other parts of the body,[27] it is very possible that the presence of LA and possibly other aPLa act with other local factors in the lower limbs leading to CLU. More studies would be required along these lines to also find out if other forms of aPLa are elevated in SCA patients with leg ulcers and to investigate whether the use of anticoagulants (oral or parenteral) would aid the healing of SCA leg ulcers. It would also be interesting to find out if patients who develop CLU secondary to other hemolytic disease apart from SCA also develop LA or/and other aPLa We conclude that SCA patients with CLU are more likely to have the LA, and this may be related to the degree of hemolysis in these patients. However, further studies are required to find out whether CLU and LA occur as a result of hemolysis or whether LA is responsible for the levels of hemolysis and CLU found in this set of patients.
